# Supplementary material for: A simple permutation‐based test of intermodal correspondence
Source: Hum Brain Mapp. 2021 Sep 14;42(16):5175–87. doi: 10.1002/hbm.25577 (PMC8519855; doi:10.1002/hbm.25577)

Supplemental figures

Figure S1: Empirical and surrogate (no. surrogates = 1000) variograms of parcellated cortical thickness measurements. These variograms are constructed to assess the reliability of the Brain Surrogate Maps with Autocorrelated Spatial Heterogeneity (BrainSMASH) method in testing for intermodal correspondence between cortical thickness and sulcal depth and cortical thickness and n-back in the left and right hemispheres (parcellations from Schaefer et al. (2018)). The horizontal axis of each figure indicates the spatial separation distance (d), and the vertical axis describes the variation between the cortical thickness measurements observed in parcels separated by distance d.

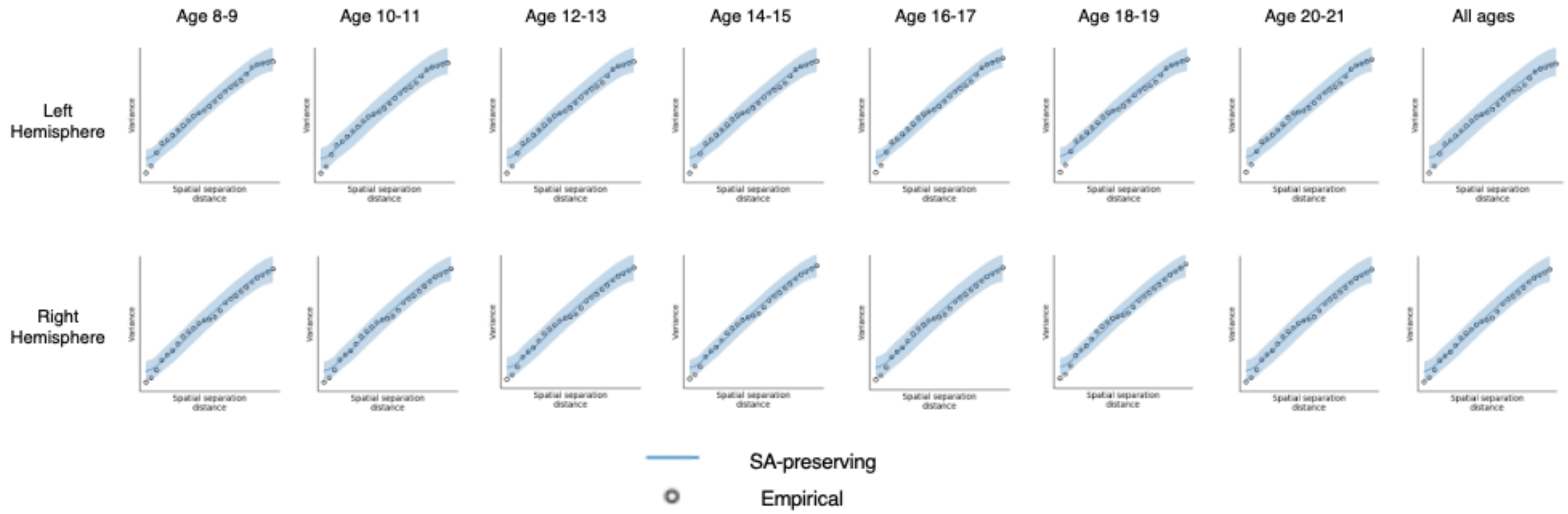

Figure S2: Null test statistic distributions corresponding to results shown in Table 1 for the SPICE test, BrainSMASH, and spin test. The observed test statistic, A_0, is plotted or written in blue. (A_0 is not plotted when it falls outside the range of the null test statistics.) Note: the observed test statistics for BrainSMASH and the spin test are not identical, even though both these methods use group-level surfaces, since the spin test removes the medial wall before calculating intermodal correspondence.

(a) Cortical thickness vs. sulcal depth (corresponds to Table 1(a)).

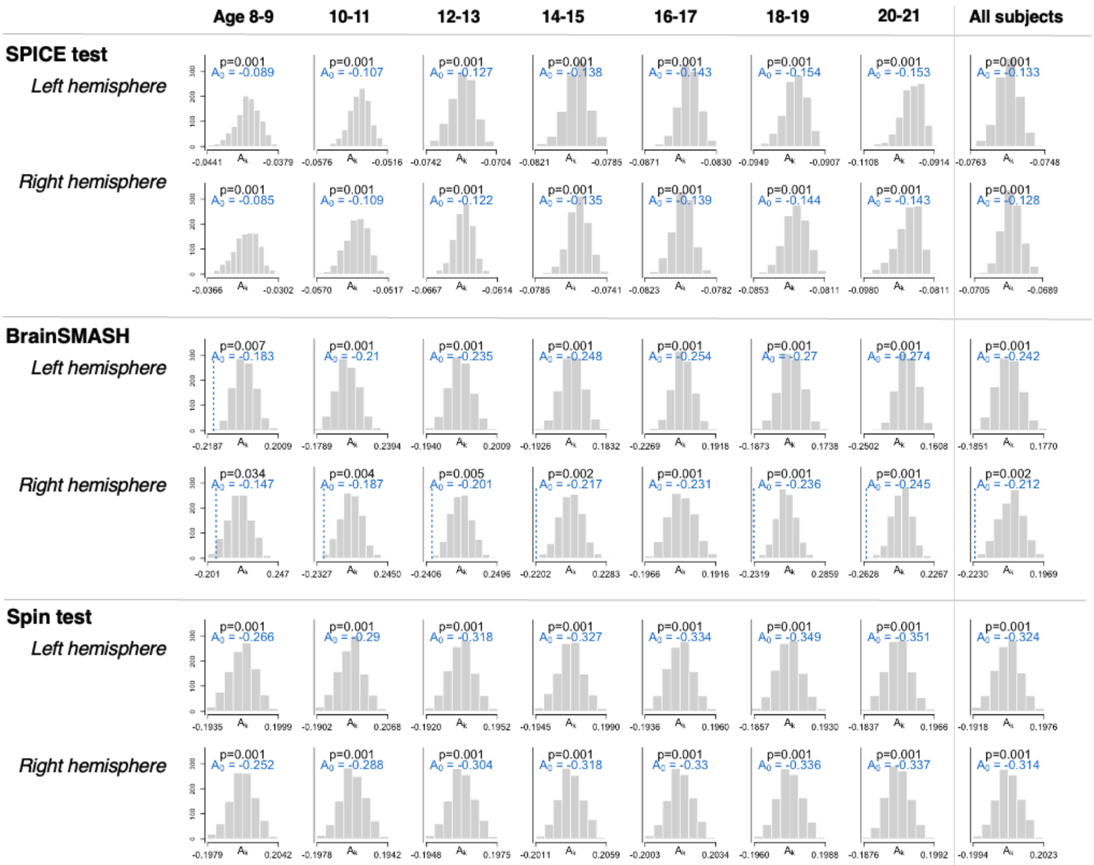

(b) Cortical thickness vs. n-back (corresponds to Table 1(b)).

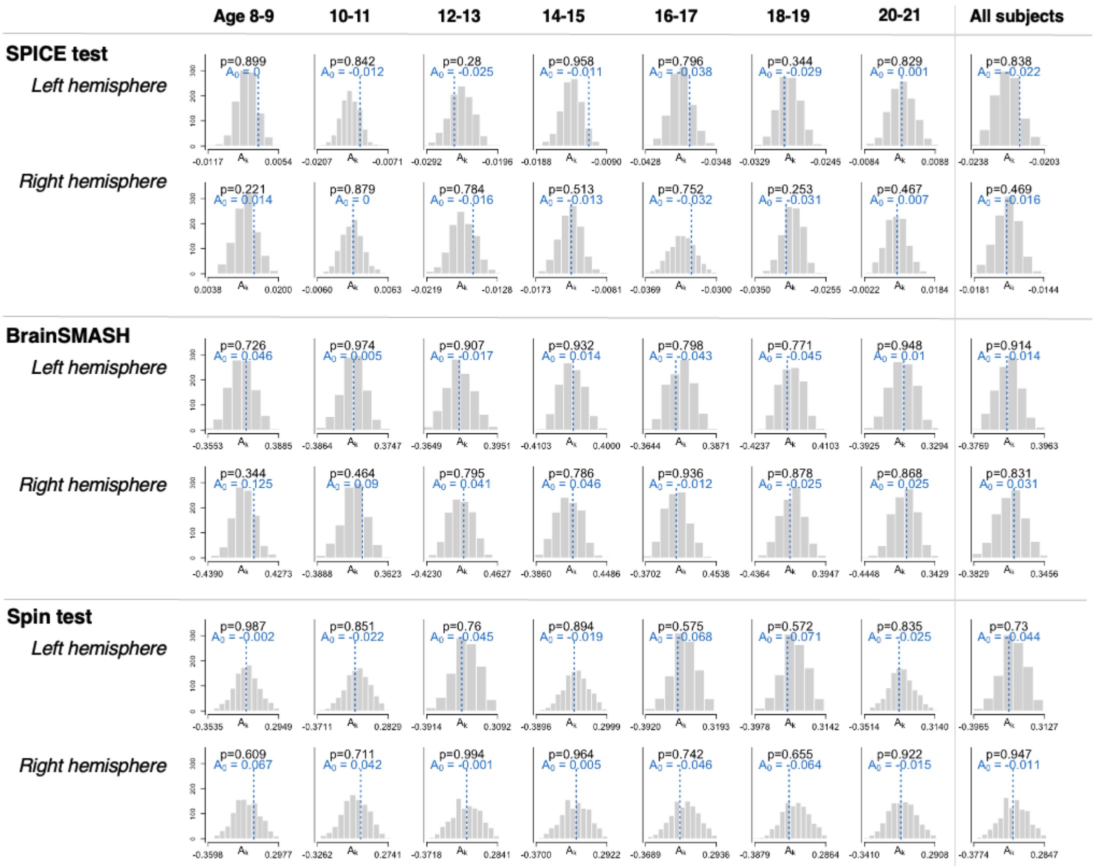

Figure S3: Empirical and surrogate (no. surrogates = 1000) variograms of parcellated cortical thickness measurements within seven functional networks, according to Yeo et al. (2011). These variograms are constructed to assess the reliability of BrainSMASH in testing for intermodal correspondence between cortical thickness and sulcal depth and cortical thickness and n-back within age-stratified groups. The horizontal axis of each plot indicates the spatial separation distance (d); the vertical axis describes the variation between cortical thickness measurements observed in parcels separated by the distance d.

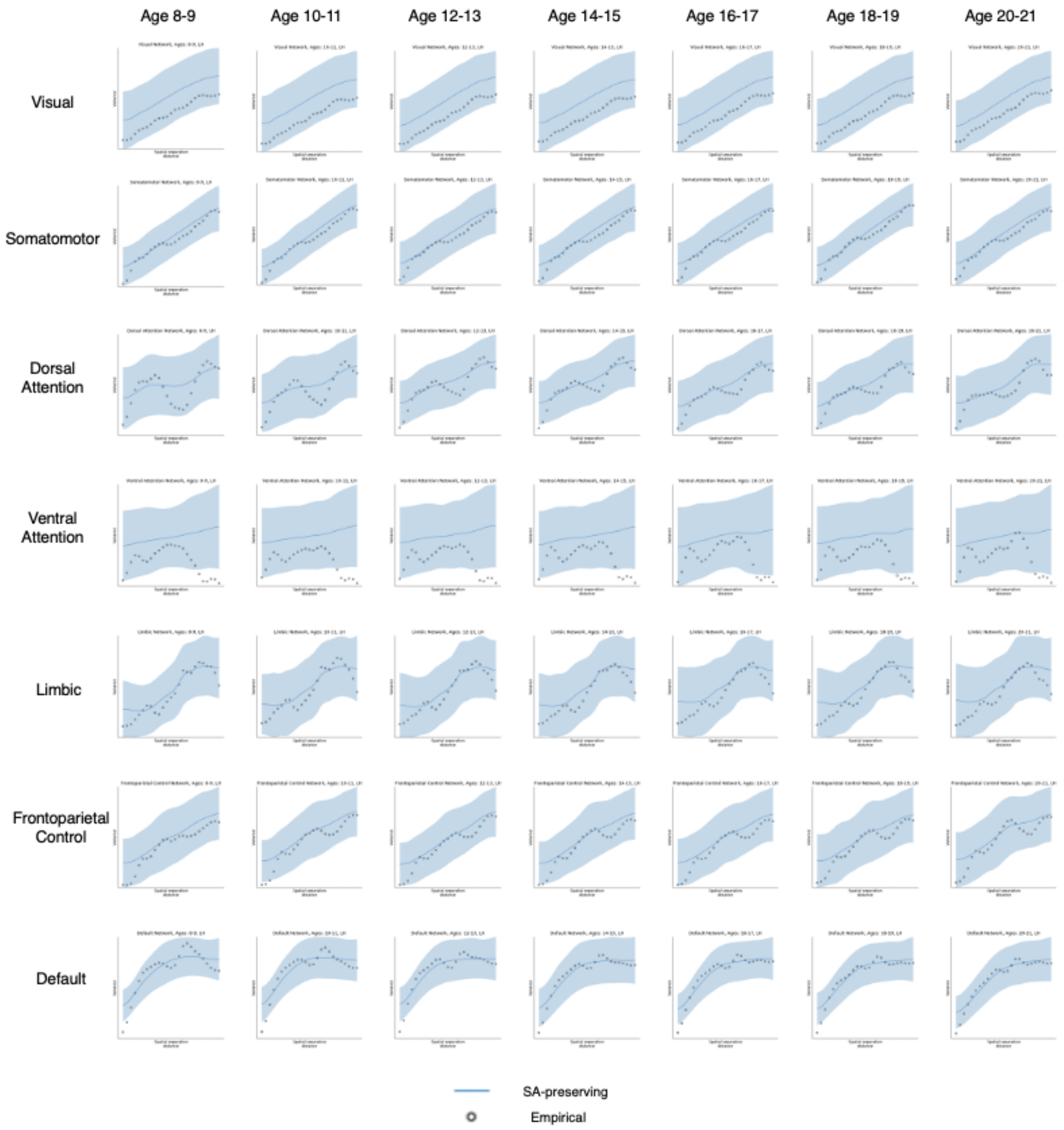

Supplement: Supplementary file 1 — FIGURE S1 Empirical and surrogate (no. surrogates = 1,000) variograms of parcellated cortical thickness measurements. These variograms are constructed to assess the reliability of the Brain Surrogate Maps with Autocorrelated Spatial Heterogeneity (BrainSMASH) method in testing for intermodal correspondence between cortical thickness and sulcal depth and cortical thickness and n‐back in the left and right hemispheres (parcellations from Schaefer et al. (2018)). The horizontal axis of each figure indicates the spatial separation distance (d), and the vertical axis describes the variation between the cortical thickness measurements observed in parcels separated by distance d FIGURE S2 Null test statistic distributions corresponding to results shown in Table 1 for the SPICE test, BrainSMASH, and spin test. The observed test statistic, A0, is plotted or written in blue (A0 is not plotted when it falls outside the range of the null test statistics). Note: the observed test statistics for BrainSMASH and the spin test are not identical, even though both these methods use group‐level surfaces, since the spin test removes the medial wall before calculating intermodal correspondence FIGURE S3 Empirical and surrogate (no. surrogates = 1,000) variograms of parcellated cortical thickness measurements within seven functional networks, according to Yeo et al. (2011). These variograms are constructed to assess the reliability of BrainSMASH in testing for intermodal correspondence between cortical thickness and sulcal depth and cortical thickness and n‐back within age‐stratified groups. The horizontal axis of each plot indicates the spatial separation distance (d); the vertical axis describes the variation between cortical thickness measurements observed in parcels separated by the distance d [file HBM-42-5175-s001.pdf]
